# DYNAMICS OF SOCIAL ISOLATION IN A NATIONAL SAMPLE OF COMMUNITY-DWELLING OLDER ADULTS

**DOI:** 10.1093/geroni/igae098.0802

**Published:** 2024-12-31

**Authors:** Mfon Umoh, Mary Louise Pomeroy, Alexandra Mueller, Taylor Craig, Katherine Ornstein, Laura Prichett, Thomas Cudjoe

**Affiliations:** Johns Hopkins University School of Medicine, Baltimore, Maryland, United States; Johns Hopkins University, Baltimore, Maryland, United States; Johns Hopkins University School of Medicine, Baltimore, Maryland, United States; Johns Hopkins University School of Medicine, Baltimore, Maryland, United States; Johns Hopkins University, Baltimore, Maryland, United States; Johns Hopkins University School of Medicine, Baltimore, Maryland, United States; Johns Hopkins University School of Medicine, Baltimore, Maryland, United States

## Abstract

Social isolation is linked to adverse health outcomes; 25% of older adults in the United States are socially isolated. However, episodic social isolation may carry different implications for health outcomes compared to chronic social isolation. Many studies examine social isolation at a single point rather than considering it as a circumstance that is intermittent or persistent. This study examined social isolation dynamics in older adults using nationally representative cohort data from 11 rounds of the National Health and Aging Trends Study (N=6,913) to describe differences between older adults with chronic, intermittent, and no social isolation. Social isolation status was derived from points given for questions about living arrangement, core discussion network size on important matters, religious attendance, and social participation. Persistent social isolation was defined as social isolation during more than 50% of available rounds and intermittent social isolation as isolation in at least one round but less than 50% of rounds. The analytic sample had social isolation scores available for a median of four rounds (IQR 3,10; M = 5.28); 20% experienced persistent isolation, 27% experienced intermittent isolation. Demographic characteristics were compared using Chi-square tests and an alpha threshold of 0.05 with the never isolated group as reference. Age, education, income, difficulty with activities of daily living, dual eligibility (Medicare and Medicaid insurance), and dementia status at baseline were correlated with social isolation persistence. Identifying factors associated with the persistence of social isolation may guide the design of interventions targeted to prevent social isolation and its detrimental consequences.

